# Operando TEM Study of Partial Oxidation of Methane Over Pd Nanoparticles

**DOI:** 10.1002/advs.202507303

**Published:** 2025-08-24

**Authors:** Yingying Jiang, Parinya (Lewis) Tangpakonsab, Alexander Genest, Günther Rupprechter, Utkur Mirsaidov

**Affiliations:** ^1^ Department of Physics National University of Singapore Singapore 117551 Singapore; ^2^ Centre for BioImaging Sciences Department of Biological Sciences National University of Singapore Singapore 117557 Singapore; ^3^ Institute of Materials Chemistry TU Wien Vienna 1060 Austria; ^4^ Centre for Advanced 2D Materials and Graphene Research Centre National University of Singapore Singapore 117546 Singapore; ^5^ Department of Materials Science and Engineering National University of Singapore Singapore 117575 Singapore

**Keywords:** DFT calculations, operando TEM, partial oxidation of methane, Pd nanocatalysts, Pd–PdO interface

## Abstract

Methane (CH_4_), which constitutes over 95% of low‐cost and abundantly available natural gas reserves, represents a key feedstock for producing syngas and other value‐added chemicals. Developing catalysts capable of efficiently converting CH_4_ into these chemicals is, therefore, crucial for reducing the dependence on limited crude oil resources. Despite the importance of these conversion reactions, the underlying details of catalyst activity remain elusive. Here, using *operando* gas‐cell transmission electron microscopy, the nanoscale mechanisms of the catalytic partial oxidation of CH_4_ over Pd nanoparticles (NPs) are explored. The observations show that the onset of the catalytic reaction directly coincides with the transformation of these NPs into robust fragmented Pd–PdO NPs. Density functional theory calculations reveal that the Pd–PdO interface plays a pivotal role in optimizing the reaction pathway: metallic Pd facilitates CH_4_ dehydrogenation, while PdO promotes C oxidation. These insights into the active structures of catalysts under working conditions provide a foundation for the rational design of high‐performance catalysts.

## Graphical abstract

This *operando* TEM study reveals that Pd nanoparticles (NPs) catalyze the partial oxidation of methane (POM) by transforming into smaller, highly dynamic mixed‐phase Pd–PdO NPs, with active sites located at the interface between the two phases. Here, metallic Pd facilitates CH_4_ dehydrogenation, while PdO promotes C oxidation. These insights provide a foundation for the rational design of high‐performance catalysts.

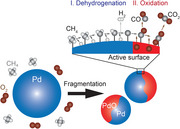

## 1 Introduction

Methane (CH_4_), the primary component of earth‐abundant natural gas, is a crucial energy source and a promising feedstock for chemical synthesis.^[^ ^1^, ^2^ ^]^ Its conversion into syngas—a mixture of H_2_ and CO—is a key step in the production of valuable liquid fuels via the Fischer–Tropsch process.^[^ ^1^ ^]^ Currently, syngas is produced through steam reforming (SRM), dry reforming (DRM), and partial oxidation (POM) reactions of methane.^[^ ^3^ ^]^ Among these, POM offers a significant advantage over the current industrial SRM process because it requires the least energy input.^[^ ^1^, ^4^ ^]^ However, the practical application of POM is hindered by persistent challenges in catalyst selectivity and stability, specifically its tendency for overoxidation of CO to CO_2_ and coke accumulation on the surface of the catalyst, respectively.^[^ ^4^, ^5^, ^6^ ^]^

Group VIII transition metals, such as Rh, Pd, Pt, and Ni, are good catalyst materials for the POM reaction, with Pd‐based systems gaining particular attention due to their high intrinsic activity.^[^ ^2^, ^4^, ^7^ ^]^ Both metallic Pd^[^ ^5^, ^8^ ^]^ and mixed‐phase Pd–PdO structures^[^ ^7^, ^9^ ^]^ have been proposed as active phases for this reaction. Under realistic flow reactor conditions, however, the composition of Pd species varies along the catalyst bed, with metallic Pd, PdO, and PdC*_x_* often coexisting.^[^ ^7^, ^10^ ^]^ This spatial heterogeneity complicates the identification of the true active sites and the elucidation of the underlying reaction mechanisms. Beyond POM, Pd–PdO catalysts are also among the most effective systems for the complete oxidation of CH_4_ to CO_2_, which is a key reaction for emission control in internal combustion engines.^[^ ^11^, ^12^, ^13^ ^]^ Although atomic‐scale understanding remains limited, several studies have suggested that an interface between Pd and PdO plays an important role in CH_4_ oxidation.^[^ ^11^, ^14^, ^15^ ^]^ Consequently, achieving high CO selectivity in POM over Pd catalysts remains an unsolved challenge, highlighting the need to understand the interplay between catalyst structure and function.

Addressing this knowledge gap requires real‐time correlation between the structure and activity of catalysts under working conditions. This is particularly important because the oxidation states^[^ ^16^, ^17^, ^18^ ^]^ and morphologies^[^ ^19^, ^20^ ^]^ of the catalysts can change rapidly in response to the reaction environment. However, the atomic‐scale details of these transformations are largely unknown.^[^ ^21^ ^]^ Recent studies using in situ spectroscopic techniques such as ambient‐pressure X‐ray photoelectron spectroscopy,^[^ ^16^ ^]^ X‐ray diffraction,^[^ ^18^ ^]^ and X‐ray absorption spectroscopy,^[^ ^18^ ^]^ have provided valuable insights into catalyst behavior, but their spatial averaging limits the ability to resolve structural changes at the level of individual nanoparticle (NP) catalysts.

To reveal the structural basis of CH_4_ oxidation and product formation, we tracked the changes in Pd NPs during the POM reaction at atmospheric pressure using *operando* gas‐cell transmission electron microscopy (TEM).^[^ ^22^, ^23^, ^24^, ^25^, ^26^, ^27^, ^28^, ^29^ ^]^ The approach enables real‐time imaging of nanocatalysts during the reaction while monitoring changes in the composition of reactants and reaction products with inline mass spectrometry.^[^ ^22^, ^23^, ^24^, ^25^, ^26^, ^27^, ^28^, ^29^ ^]^

## 2 Results and discussion

**Figure 1:**
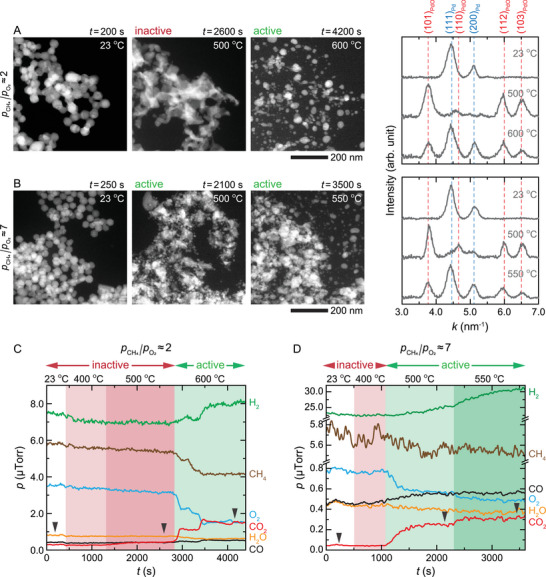
Transformations in Pd NPs during the POM reaction. Scanning TEM (STEM) images and electron diffraction profiles (Figure S4, Supporting Information) of Pd NPs during the POM reaction at A) $p_{CH_{4}}/p_{O_{2}}\approx 2$ (760 Torr of 17% CH_4_, 10% O_2_, and 73% Ar) and B) $p_{CH_{4}}/p_{O_{2}}\approx 7$ (760 Torr of 22% CH_4_, 3% O_2_, and 75% Ar). Corresponding changes in the reactant (CH_4_ and O_2_) and product (H_2_, CO, and CO_2_) gas compositions during the POM reaction at C) $p_{CH_{4}}/p_{O_{2}}\approx 2$ and D) $p_{CH_{4}}/p_{O_{2}}\approx 7$. The high background signals of H_2_, H_2_O, and CO were from ionization reactions of CH_4_ and O_2_ in the mass spectrometer (see SI Section 1 for more details). The dashed vertical lines in the diffraction profiles correspond to the diffraction peaks of Pd (blue) and PdO (red). The black arrows in (C, D) correspond to the time points of the STEM image series and electron diffraction profiles shown in (A, B).

**Figure** **1** shows the morphological evolution of Pd NPs under a reactive gas mixture of CH_4_ and O_2_ at an atmospheric pressure (760 Torr). At $p_{CH_{4}}/p_{O_{2}}\approx 2$ (760 Torr of 17% CH_4_, 10% O_2_, and 73% Ar), the as‐synthesized metallic NPs were fully oxidized into PdO at 500 °C (Figure 1A: *t* = 200–2600 s). These PdO NPs did not catalyze the POM reaction, as evidenced by the lack of product formation (Figure 1C: *t* = 1300–2800 s). However, upon increasing the temperature to 600 °C, the PdO NPs decomposed into smaller NPs and started to catalyze the production of CO_2_ and H_2_ (Figure 1A: *t* = 4200 s and Figure 1C: *t* = 2800–4400 s). Electron diffraction patterns reveal that the smaller active NPs comprise the Pd and PdO phases (i.e., Pd–PdO NPs).

Next, to explore the effect of gas composition, in a similar fashion, we tested the Pd NPs under an O_2_‐poor condition with $p_{CH_{4}}/p_{O_{2}}\approx 7$ (760 Torr of 22% CH_4_, 3% O_2_, and 75% Ar). At 500 °C, the Pd NPs transformed into the active Pd–PdO phase, producing H_2_ and a mixture of CO_2_ and CO, with CO_2_ and CO accounting for 80% and 20% of this mixture, respectively (Figure 1B: *t* = 2100 s, Figure 1D: *t* = 1100–2300 s, and Figure S2B, Supporting Information). Furthermore, the shapes and positions of these NPs underwent significant changes at 500–550 °C (Figure 1B: *t* = 2100–3500 s).

The NPs exhibited different oxidation states in the two distinct atmospheres at 500 °C (i.e., PdO at $p_{CH_{4}}/p_{O_{2}}\approx 2$ vs Pd–PdO at $p_{CH_{4}}/p_{O_{2}}\approx 7$) (Figure 1A,B). This indicates that the mixed‐phase Pd–PdO is more active than the pure PdO phase (Figure 1C: *t* = 1300–2800 s vs Figure 1D: *t* = 1100–2300 s). In fact, running the reaction with Pd–PdO NPs under $p_{CH_{4}}/p_{O_{2}}\approx 2$ atmosphere at 500 °C, catalyzes the CH_4_ oxidation reaction, further validating the high catalytic activity of the mixed phase (Figure S3, Supporting Information).

In our *operando* tests, the primary products of the CH_4_ oxidation reaction were CO_2_ and H_2_ (Figure 1C,D). This is different from the typical products obtained in conventional flow reactor studies, where the products are CO and H_2_ at $p_{CH_{4}}/p_{O_{2}}\geq 2$:^[^ ^1^, ^9^ ^,^ ^30^ ^]^

(1) $$CH_{4}+\frac{1}{2}O_{2}\to \mathrm{CO}+2H_{2}\;\Delta H_{r}^{o}\left(298\;K\right)=-36\;\mathrm{kJ}\;\mathrm{mo}l^{-1}$$

This difference likely arises from the presence of residual O_2_ in the reactive gas atmosphere inside the gas cell. In conventional flow reactors, reactant gases pass slowly through a catalyst‐filled tube, with residence times ranging from tens to hundreds of milliseconds,^[^ ^31^ ^]^ allowing for nearly complete O_2_ consumption.^[^ ^9^, ^30^ ^]^ In contrast, during *operando* TEM studies, the residence times within the small reaction zone of the gas cell are significantly shorter (i.e., <1 ms),^[^ ^23^ ^]^ leading to incomplete conversion of reactants and residual CH_4_ and O_2_ (Figure 1C,D). As a result of this residual O_2_, Pd NPs further oxidize the CO product into CO_2_ ($\mathrm{CO}+\frac{1}{2}O_{2}\to CO_{2}$).^[^ ^25^, ^32^, ^33^, ^34^ ^]^ Although the H_2_O production was not detected in our study, the possibility of H_2_ oxidation ($H_{2}+\frac{1}{2}O_{2}\to H_{2}O$) and CH_4_ combustion ($CH_{4}+2O_{2}\to CO_{2}+2H_{2}O$) cannot be excluded,^[^ ^35^, ^36^ ^]^ as minute amounts of water products may have easily condensed on the walls of the outlet tube. Our observation, showing a strong CO_2_ signal, is consistent with findings from conventional reactor studies, which report CO_2_ as the dominant product at low conversion rates of CH_4_ and O_2_, and CO as the major product at higher conversion rates.^[^ ^9^, ^10^, ^30^ ^]^

**Figure 2:**
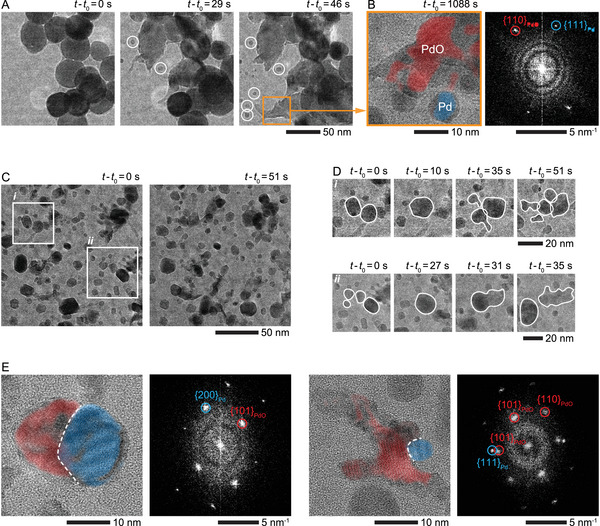
Fragmentation and restructuring of Pd NPs during the POM reaction. A) In situ TEM image series showing the fragmentation of Pd NPs at $p_{CH_{4}}/p_{O_{2}}\approx 7$ (760 Torr of 22% CH_4_, 3% O_2_, and 75% Ar) and at 500 °C (Video S1, Supporting Information). The white circles indicate small NPs that formed through the fragmentation of the original NPs. *t*_0_ is the time point at which the NPs were heated to 500 °C. B) The enlarged view of the region (orange box) in (A) and its FFT pattern showing that the NPs were oxidized to PdO, and the newly formed small NPs were metallic Pd. C) In situ TEM image series of the NPs at $p_{CH_{4}}/p_{O_{2}}\approx 7$ and at 500 °C (Video S2, Supporting Information). *t*_0_ is the time point at which we started recording the process. D) The two enlarged views of the regions *i* and *ii* in (C) show the coalescence and splitting of the NPs. E) TEM images and FFT patterns of two NPs at $p_{CH_{4}}/p_{O_{2}}\approx 7$ and at 500 °C. The dashed white curves indicate the Pd–PdO interfaces in the NPs. The inverse FFT patterns, highlighting Pd (blue) and PdO (red) regions, are overlaid on the TEM images in (B, E).

To better understand how the active Pd–PdO phase emerges, we tracked the evolution of Pd NPs during the reaction with TEM (**Figure** **2**). Figure 2A shows how the original Pd NPs changed when they were heated to 500 °C in the reactive environment at $p_{CH_{4}}/p_{O_{2}}\approx 7$. First, the Pd NPs increase in size due to oxidation, and then parts of these NPs fragment, producing smaller NPs around the original ones (Figure 2A: *t* – *t*_0_ = 0–46 s). A closer inspection of the process revealed that these smaller NPs are pure Pd NPs, with the fragmentation occurring during the reduction of the oxidized NPs (Figure 2B). The simultaneous oxidation and reduction of the catalysts are due to the presence of both oxidizing (i.e., O_2_^[^ ^37^, ^38^, ^39^ ^]^) and reducing (i.e., CH_4_,^[^ ^38^, ^39^ ^]^ H_2_,^[^ ^38^, ^39^ ^]^ and CO^[^ ^37^ ^]^) gases in the reactive atmosphere.

As the conversion reaction progresses, the small fragmented NPs display very dynamic behavior (Figure 2C; Video S2, Supporting Information), such as continuous coalescence (Figure 2D: *i*) *t* – *t*_0_ = 0–10 s and *ii*) *t* – *t*_0_ = 0–27 s) and splitting (Figure 2D: *i*) *t* – *t*_0_ = 10–51 s and *ii*) *t* – *t*_0_ = 27–35 s). Moreover, the high‐resolution TEM images of the active NPs taken in the midst of the reaction reveal that these dynamic NPs comprise Pd and PdO domains separated by a distinct interface (Figure 2E). Based on our observations, the dynamic changes (i.e., coalescence and splitting) of the NPs help them maintain their active Pd–PdO phase and small size (with a high surface‐to‐volume ratio) needed to prevent loss of activity associated with coalescence (i.e., decrease of surface‐to‐volume ratio) (Video S2, Supporting Information).

**Figure 3:**
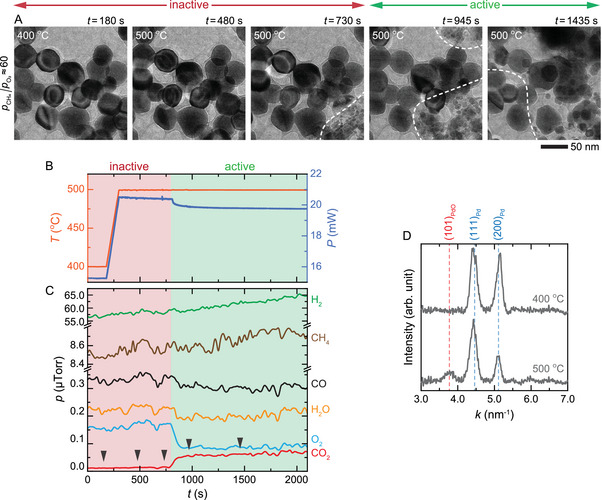
Fragmentation of Pd NPs and ignition of the POM reaction. A) In situ TEM image series of the NPs, B) heater power and the corresponding measured temperature, and C) changes in the amount of reactant and product gases during the POM reaction at $p_{CH_{4}}/p_{O_{2}}\approx 60$ (760 Torr of 60% CH_4_, 1% O_2_, and 39% Ar) and at 400–500 °C (Video S3, Supporting Information). Note that despite the excessive amount of CH_4_, residual O_2_ still drives CO overoxidation, making CO_2_ the main product. D) Electron diffraction profiles of the NPs before (400 °C) and after (500 °C) fragmentation showing that Pd NPs transformed into Pd–PdO NPs (Figure S5, Supporting Information). The dashed white curves in (A) indicate the regions with the fragmented NPs. The black arrows in (C) correspond to the time points of the TEM image series shown in (A). The dashed vertical lines in (D) correspond to the diffraction peaks of Pd (blue) and PdO (red).

While our *operando* TEM results establish the Pd–PdO NPs as the active nanostructures and that PdO phase alone doesn't catalyze the POM reaction (Figures 1 and 2), it doesn't rule out the possibility that pure Pd may also be an active catalyst. To test whether the pure Pd phase can catalyze the reaction, we monitored how Pd NPs evolve under highly reducing conditions, $p_{CH_{4}}/p_{O_{2}}\approx 60$ (760 Torr of 60% CH_4_, 1% O_2_, and 39% Ar), where the oxidation of Pd NPs is expected to proceed slowly, giving us ample time to monitor the changes carefully.^[^ ^23^, ^25^ ^]^ Here, most NPs were covered by a layer of amorphous material that likely formed from the decomposition of CH_4_ into carbonaceous products on the Pd surface. Initially, as the temperature reached 500 °C, the Pd NPs remained unchanged and showed no catalytic activity (**Figure** **3**A–C: *t* = 480 s). Over time, the NPs began to fragment into small Pd–PdO NPs (Figure 3A: *t* = 730–1435 s and Figure 3D), and the catalytic activity was first detected during this fragmentation process (Figure 3C: *t* = 800–2100 s). The ignition point of the exothermic POM reaction, marked by a 0.5 mW drop in the heater power at *t* = 800 s (Figure 3B),^[^ ^33^ ^]^ lagged the fragmentation onset at *t* = 730 s (Figure 3A), indicating that a detectable amount of gas products was produced only when a sufficient number of NPs were transformed into small Pd–PdO NPs (Figure 3A–D). These results align with the observations at $p_{CH_{4}}/p_{O_{2}}\approx 2$ and ≈ 7 (Figure 1), with $p_{CH_{4}}/p_{O_{2}}\approx 7$ being favourable for Pd NPs turning active to catalyze the POM reaction because of the rapid formation of Pd–PdO phase under moderately oxidizing conditions. Hence, it is the Pd–PdO interface that catalyzes CH_4_ oxidation.

To understand why a mixed‐phase Pd–PdO system is needed for catalyzing the conversion reaction, one must consider the role of each phase in the reaction. From the atomistic perspective, the CH_4_ oxidation pathway on Pd catalysts involves dehydrogenation and oxidation steps.^[^ ^15^ ^]^ CH_4_ first dehydrogenates into CH_3_^*^, CH_2_^*^, and other carbonaceous species, which are then oxidized into CO and CO_2_. Metallic Pd is more effective than PdO at dehydrogenation of CH_4_ (e.g., the activation barriers for CH_4_ dissociation are 0.6–1.0 eV on Pd surfaces^[^ ^40^, ^41^, ^42^ ^]^ and 1.4–1.6 eV on PdO surfaces^[^ ^41^, ^43^ ^]^), whereas PdO is more efficient at oxidizing carbonaceous intermediates.^[^ ^40^, ^44^ ^]^ This is consistent with our observations, which show that the catalytic reactions predominantly occur in the presence of PdO, and amorphous carbon accumulates on metallic Pd (Figure 3). Therefore, we propose the reaction pathway at the Pd–PdO interface that leverages the distinct advantages of both Pd and PdO phases.

**Figure 4:**
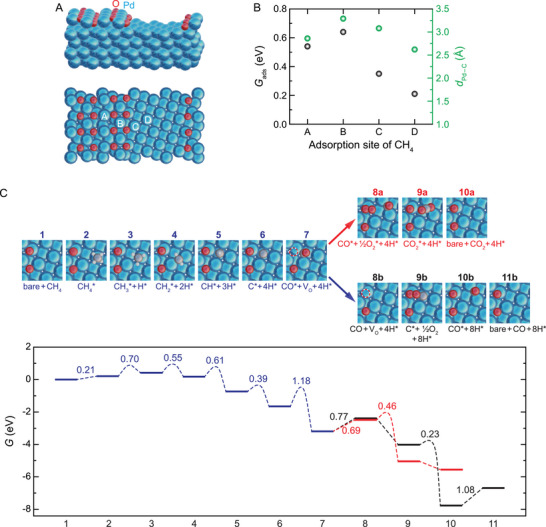
CH_4_ oxidation reaction pathway at the Pd–PdO interface. A) Side and top views of the Pd–PdO interface model. The model is composed of a supporting Pd(100) surface and a partial PdO(101) monolayer, which contains Pd^2+^ and Pd^1+^ species (Table S2 and Figure S7, Supporting Information). B) CH_4_ adsorption free energies (*G*_ads_) and the Pd–C (of CH_4_) distance (*d*_Pd − C_) for the four adsorption sites indicated in (A). C) (Top) Atomic configurations and the (bottom) corresponding free energy (*G*) diagram for the CH_4_ oxidation reaction at the Pd–PdO interface (see Figure S8B, Supporting Information for enlarged views and additional transition states). The reaction involves CH_4_ dehydrogenation to elemental C^*^ (steps 1→6) and oxidation of C^*^ to CO^*^ (step 6→7) (blue lines), followed by the formation of CO_2_ (steps 7→10a) (red lines) or CO (steps 7→11b) (black lines). Activation free energies (*G*_a_) and the change in free energies (Δ*G*) of reaction steps are listed in the plot (see Tables S4 and S5, Supporting Information for more details). ^*^ represents surface‐bound (adsorbed) species.

To test this hypothesis, we used density functional theory (DFT) calculations to model CH_4_ oxidation at the Pd–PdO interface. To mimic our experimental conditions, the free energies (*G*) during the reactions were evaluated at 800 K and 1 atm. **Figure** **4**A shows the Pd–PdO interface model, consisting of a Pd(100) substrate partially covered with a PdO(101) monolayer. First, CH_4_ adsorption was examined at four sites: the PdO region (sites A and B), the Pd–PdO interface (site C), and the Pd region (site D) (Figure 4A). The adsorption processes at all four sites are endothermic, with site D exhibiting the lowest thermodynamic cost and the shortest Pd─C (of CH_4_) bond length (Figure 4B; Figure S8A, Supporting Information). Additionally, the dissociated CH_4_ species (i.e., CH_3_^*^, CH_2_^*^, CH^*^, and C^*^) bind strongly to the Pd region near the interface (Table S3, Supporting Information). These results show that the metallic Pd region exhibits a stronger affinity toward CH_4‐_ *_x_* ^*^ species compared to the PdO region, indicating that dissociation and dehydrogenation of CH_4_ occur on the Pd region of the Pd–PdO interface.

We then calculated the full reaction pathway for CH_4_ oxidation at the Pd–PdO interface (Figure 4C). Following CH_4_ adsorption onto the Pd surface (step 1→2), the dehydrogenation steps (steps 2→6) proceed with low activation energy barriers (*G*_a_ ≤ 0.70 eV) on the Pd region. The final product of these steps, C^*^, resides on the hollow site of the Pd surface,^[^ ^45^ ^]^ positioned adjacent to the Pd–PdO boundary (state 6). For simplicity and in line with earlier computational studies,^[^ ^15^, ^40^ ^]^ we chose the sequential dehydrogenation of CH_4_ into C^*^ as the reaction pathway on the Pd surface. However, it is worth noting that alternative reaction pathways involving oxygenated hydrocarbons on the surface may also be present.^[^ ^20^, ^40^, ^44^ ^]^ Notably, on a pure Pd surface (without PdO), the oxidation of C^*^ is the rate‐limiting step for CH_4_ oxidation with a high energy barrier (e.g., 1.81 eV on Pd(100)).^[^ ^40^ ^]^ However, at the Pd–PdO interface, this barrier significantly reduces (*G*_a_ = 1.18 eV) as C^*^ directly reacts with a neighboring lattice O atom to form CO^*^, creating an oxygen vacancy (V_O_) (step 6→7). CO^*^ can then oxidize into CO_2_^*^ by reacting with the O_2_^*^ filled at the V_O_ site (steps 7→9a), followed by CO_2_ desorption from the interface (step 9a→10a). Alternatively, CO^*^ may desorb directly into the gas phase (step 7→8b). In this case, the V_O_ is subsequently replenished by O_2_, facilitating further oxidation of C^*^ into CO^*^ (steps 8b→10b). These findings align with our experimental results, which show high activity of Pd–PdO NPs for converting CH_4_ into CO_2_ and CO (i.e., the production of CO_2_ in Figures 1C,D and 3C, as well as the production of a small amount of CO in Figure 1D).

Previous studies of Pd catalysts in POM and CH_4_ combustion reactions, which share similar elemental steps,^[^ ^40^ ^]^ have attributed the activity to metallic Pd,^[^ ^46^, ^47^ ^]^ PdO,^[^ ^12^, ^17^ ^]^ or a mixture of Pd and PdO, including PdO*_x_* ^[^ ^15^ ^]^ and PdO^[^ ^13^, ^19^, ^20^ ^]^ layers formed on metallic Pd. However, the atomistic mechanism and the precise role of the Pd–PdO interface in the catalytic process remained unclear. Our findings demonstrate that the coexistence of Pd and PdO phases optimizes the reaction pathway by enabling dehydrogenation at the Pd region and oxidation with lattice O atoms from the PdO region. Although our *operando* observations directly identified only crystalline Pd and PdO phases, the dynamic phase transition between them may generate small amounts of transiently nonstoichiometric PdO*_x_* species. Even if such species are present, previous simulations have shown that they consist of intermixed Pd and PdO regions, closely resembling the Pd–PdO interfaces observed in our study.^[^ ^15^ ^]^ It is worth noting, in contrast to earlier studies by Yue et al.^[^ ^19^ ^]^ and Tang et al.,^[^ ^20^ ^]^ who only examined the combustion of methane, our study clearly describes the detailed mechanisms of partial oxidation of methane, a reaction pathway that is crucial for the downstream synthesis of value‐added reaction products.

**Figure 5:**
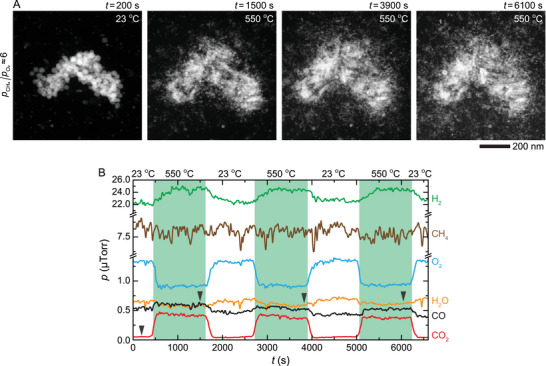
Three cycles of the POM reaction. A) In situ STEM image series of the NPs and B) corresponding changes in the amount of reactant and product gases during the POM reaction at $p_{CH_{4}}/p_{O_{2}}\approx 6$ (760 Torr of 25% CH_4_, 4% O_2_, and 71% Ar) and at 23–550 °C. The black arrows correspond to the time points of the image series shown in (A).

The hallmark of an effective catalyst is not only its high activity but also its stability. To establish whether the Pd–PdO NPs are stable, and if so, to identify the reasons for their stability, we tracked the active Pd–PdO NPs during three consecutive reaction cycles at 23–550 °C (**Figure** **5**). The activity of the NPs remained stable, as the fragmented NPs maintained good dispersion throughout the extended cumulative reaction time (Figure 5A,B: *t* = 1500 s vs *t* = 6100 s). The reason behind such dispersion is their highly dynamic and continuous restructuring (i.e., coalescence followed by immediate fragmentation), as discussed in Figure 3C,D, which prevents their sintering and carbon poisoning.

Finally, it is important to note that our approach uses a simplified model system of pure, unsupported Pd NPs inside gas‐cell microreactors, which may not fully replicate catalytic conditions in conventional reactors. While this may result in deviations in the amount of reaction products, these *operando* studies effectively capture the essential features of active catalyst structures during the reaction. Hence, these observations provide a good starting point for future studies to elucidate the atomic‐scale details of catalytic activity. Such insights are necessary to address numerous open questions, including the origin of selectivity for CO production over CO_2_ under various reaction conditions, which remains unresolved.

## 3 Conclusion

Our study shows that Pd–PdO NPs catalyze the CH_4_ oxidation at the interface, with Pd facilitating CH_4_ dehydrogenation and PdO supplying lattice oxygen for carbon oxidation. The continuous restructuring of the NPs during the reaction not only prevents the sintering of the NP catalysts but also exposes new active sites for the reaction, thereby maintaining catalytic stability. More generally, this direct approach to observing a catalytic reaction will provide much‐needed insight into dynamic catalytic processes and aid in the design of efficient catalysts for a broad range of chemical processes.

## 4 Experimental Section

### Materials

The following reagents from Sigma–Aldrich Co. (St Louis, MO, USA) were used to synthesize the Pd NPs: palladium(II) chloride (PdCl_2_, Cat. No. 323373), L‐ascorbic acid (C_6_H_8_O_6_, Cat. No. A7506), hexadecyltrimethylammonium bromide (CTAB, Cat. No. H9151), sodium iodide (NaI, Cat. No. 409286), and hydrochloric acid (HCl, Cat. No. 258148). Deionized (DI) water with a resistivity of 18.2 MΩ cm was used to prepare all aqueous solutions used in this study.

### Synthesis of Pd NPs

The Pd NPs were synthesized using a seed‐mediated growth method described by Niu et al.^[^ ^48^ ^]^ with some modifications. First, a 10 mm H_2_PdCl_4_ solution was prepared by dissolving 18 mg of PdCl_2_ in 10 mL of 20 mm HCl solution. Then, 1.25 mL of 100 mm CTAB solution, 0.5 mL of 10 mm H_2_PdCl_4_ solution, and 8.75 mL of DI water were mixed in a 20 mL glass vial. The vial containing the solution was heated at 95 °C in an oil bath with stirring. After 5 min, 160 µL of 100 mm ascorbic acid solution was added to the pre‐heated solution, and the mixed solution was kept at 95 °C for 20 min. These synthesized NPs were used as seeds.

In the subsequent stage of Pd NP growth, 25 mL of 100 mm CTAB and 25 µL of 10 mm NaI solutions were mixed and kept at 80 °C in a water bath. Next, 625 µL of 10 mm H_2_PdCl_4_, 5 mL of Pd seed, and 25 µL of 100 mm ascorbic acid solutions were added to the pre‐heated solution. The mixed solution was kept at 80 °C for 60 min. The synthesized NPs were washed four times by centrifugation with DI water before use.

### *Operando* TEM Experiments

A 300‐kV Thermo Fisher Titan (S)TEM (Thermo Fisher Scientific Inc., Hillsboro, OR, USA) equipped with a Gatan K2 IS camera (Gatan Inc., Pleasanton, CA, USA) was used for *operando* TEM studies. In situ TEM image series were recorded at a rate of 5 frames per second with an electron flux of 100−200 e^−^ Å^−2^ s^−1^.

A DENSsolutions Climate TEM holder (DENSsolutions, Delft, Netherlands) was used for *operando* TEM studies of the NPs in reactive gaseous environments. Prior to the experiments, 5 µL of the Pd NP solution was drop‐cast onto the bottom chip of a gas cell (DENSsolutions, Delft, Netherlands) and allowed to dry.^[^ ^25^, ^33^ ^]^ The gas cell was then assembled and checked for leaks before being inserted into the TEM. For all *operando* TEM studies, gases were introduced into the cell at a flow rate of 40−50 µL min^−1^ and a pressure of 760 Torr using the DENSsolutions gas delivery system. Simultaneously, the outlet gas from the holder was analyzed using a quadrupole mass spectrometer (Stanford Research Systems, Sunnyvale, CA, USA) with the pressure of the gas passing through the analyzer chamber maintained at ≈10^−5^ Torr.

To facilitate interpretation of the mass spectrometry profiles shown in Figures 1, 5, and 5B, several clarifications are provided. The CH_4_ signal appears noisier in Figure 1D than in Figure 1C simply because the *y*‐axis in Figure 1D was expanded to better visualize small changes in all gas compositions. In Figure 3C, after ignition, the slight increase in the amount of CH_4_ was due to gas feed instability, while the drop in the CO signal was likely due to the increase in the total amount of gas molecules, which dilutes the CO partial pressure. Regarding the nearly constant CH_4_ signal in Figure 5B, this was consistent with previous *operando* studies,^[^ ^22^, ^23^, ^33^ ^]^ and was due to the low CH_4_ conversion rate (≈ 5%), where subtle changes were likely masked by signal noise.

### Plotting the Electron Diffraction Profiles

The radial diffraction profiles in Figures 1, 3 and Figure S3A (Supporting Information) were the plots of the summed intensity, *S*(*k*) = ∑*I*(***k***), obtained from electron diffraction images after the background subtraction. Here, ***k*** = (*k_x_*, *k_y_*) and $k=\sqrt{k_{x}^{2}+k_{y}^{2}}$ are the outward radial vector from the center of the diffraction pattern and its corresponding length, and *I*(***k***) is the diffraction intensity at that point.

### Quantification of CO_2_ and CO Products

The amount of CO_2_ and CO in different atmospheres (e.g., $p_{CH_{4}}/p_{O_{2}}\approx 2$ and $p_{CH_{4}}/p_{O_{2}}\approx 7$), as shown in Figure 1C,D, were estimated and plotted in Figure S2 (Supporting Information). First, the background signals of CO_2_ ($p_{\mathrm{base}\_CO_{2}}$) and CO ($p_{\mathrm{base}\_\mathrm{CO}}$) were obtained by averaging their partial pressures ($p_{CO_{2}}$ and *p*_CO_) before the onset of the catalytic reaction. During the reaction, the net partial pressures of CO_2_ ($p_{\mathrm{net}\_CO_{2}}$) and CO ($p_{\mathrm{net}\_\mathrm{CO}}$) were determined as follows:

(2) $$p_{\mathrm{net}\_CO_{2}}=p_{CO_{2}}-p_{\mathrm{base}\_CO_{2}}$$

(3) $$p_{\mathrm{net}\_\mathrm{CO}}=p_{\mathrm{CO}}-p_{\mathrm{base}\_\mathrm{CO}}-10\%\times p_{\mathrm{net}\_CO_{2}}$$

The term $10\%\times p_{\mathrm{net}\_CO_{2}}$ accounts for the CO signal arising from CO_2_ fragmentation during electron ionization in the mass spectrometer, which is around 10%.^[^ ^49^ ^]^

Subsequently, the percentages of CO_2_ and CO were calculated as follows:

(4) $$CO_{2}\%=\frac{p_{\mathrm{net}\_CO_{2}}}{p_{\mathrm{net}\_CO_{2}}+p_{\mathrm{net}\_\mathrm{CO}}}\times 100\%$$

(5) $$\mathrm{CO}\%=\frac{p_{\mathrm{net}\_\mathrm{CO}}}{p_{\mathrm{net}\_CO_{2}}+p_{\mathrm{net}\_\mathrm{CO}}}\times 100\%$$

### DFT Computations

Spin‐polarized DFT calculations were performed using the Vienna Ab initio Simulation Package (VASP) v6.2.1,^[^ ^50^, ^51^ ^]^ employing the projector augmented‐wave (PAW) method.^[^ ^52^ ^]^ Exchange‐correlation energies were approximated within the generalized gradient approximation (GGA) as parameterized by Perdew, Burke, and Ernzerhof (PBE).^[^ ^53^ ^]^ The cutoff energy for the plane‐wave basis set was 450 eV, and the Brillouin zone integration was carried out using a 1×2×1 *k*‐point grid. A Hubbard model based on the Dudarev formalism (DFT+*U*)^[^ ^54^ ^]^ was applied to describe oxidized Pd centers in the PdO region. The model corrects for the self‐interaction error (SIE) in systems with strongly correlated *d*‐ and *f*‐electrons, a common issue in transition metal oxides.^[^ ^55^, ^56^ ^]^ The introduction of the *U* parameter may slightly influence activation barriers on PdO surfaces compared to results without it (e.g., < 0.02 eV).^[^ ^17^ ^]^ Here, the effective *U* parameter was set to 6.5 eV, which accurately reproduces the experimental energy band gap and lattice constant of bulk PdO (Figure S6, Supporting Information).

Van der Waals (vdW) interactions were incorporated using the Grimme (DFT‐D3) method with Becke–Johnson damping (Table S3, Supporting Information).^[^ ^57^ ^]^ Transition states were localized using the climbing image nudged elastic band (CI‐NEB) method.^[^ ^58^, ^59^ ^]^ The convergence criterion for geometry optimization was set to 0.05 eV Å^−1^. Vibrational modes were calculated using a finite‐difference approach with a displacement width of 0.015 Å in VASP. All transition states were confirmed to have exactly one imaginary mode. Thermodynamic quantities for free energy calculations were evaluated using the Thermochemistry module of the Atomic Simulation Environment (ASE).^[^ ^60^ ^]^

The Gibbs free energies (*G*), shown in Figure 4B,C and Tables S3–S5 (Supporting Information), were calculated at 800 K and 1 atm of each gas‐phase species, mimicking experimental conditions (Figures 1, 2, 3 and 5). For adsorbates, all degrees of freedom were considered in a harmonic limit, excluding frustrated rotational and translational motions. For gas‐phase species, translational, rotational, and vibrational motions were approximated in an ideal gas limit at 1 atm. Vibrational frequencies below 100 cm^−1^ were set to 100 cm^−1^. The adsorption free energies (*G*_ads_) and free energies of the reactions (Δ*G*) were calculated using the following equations:

(6) $$G_{\mathrm{ads}}=G_{\mathrm{complex}}-\left(G_{\mathrm{surface}}+G_{\mathrm{gas}}\right)$$

(7) $$\Delta G=G_{\mathrm{product}}-G_{\mathrm{reactant}}$$

where *G*_complex_, *G*_surface_ and *G*_gas_ represent the free energies of the Pd–PdO surface with adsorbates, the bare Pd–PdO surface (taken from DFT total energy^[^ ^61^ ^]^), and the gas phase, respectively. *G*_reactant_ and *G*_product_ are the free energies of the reactant and product states, respectively. The activation free energies (*G*_a_) were calculated as:

(8) $$G_{a}=G_{\mathrm{TS}}-G_{\mathrm{reactant}}$$

where *G*_TS_ is the free energy at a transition state. Additionally, adsorption energies (*E*_ads_), reaction energies (Δ*E*), and activation energies (*E*_a_) from DFT total energies (at 0 K and vacuum) were calculated in the same fashion (Tables S3–S5, Supporting Information).

### Computational Model

The Pd–PdO interface model shown in Figure 4A was built based on the work of Kinnunen et al.^[^ ^14^ ^]^ The model consisted of four layers of Pd(100) and a partial monolayer of PdO(101). A 12 Å vacuum was added perpendicular to the surface. This configuration was chosen because both the Pd(100) and PdO(101) facets are active for the CH_4_ oxidation reaction,^[^ ^40^, ^44^ ^]^ and the PdO(101) layers were stable on the Pd(100) surface.^[^ ^62^ ^]^ The partial PdO(101) monolayer contains both Pd^1+^ and Pd^2+^ species (Figure S7 and Table S2, Supporting Information). During structural optimization of the model, the two lowest Pd layers were kept at their bulk lattice parameters while the remaining atoms were allowed to move.

## Conflict of Interest

The authors declare no conflict of interest.

## Supporting information



Supporting Information

Supplemental Video 1

Supplemental Video 2

Supplemental Video 3

## Data Availability

The data that support the findings of this study are available in the supplementary material of this article.
