# Supplementary material for: Operando TEM Study of Partial Oxidation of Methane Over Pd Nanoparticles
Source: Adv Sci (Weinh). 2025 Aug 24;12(43):e07303. doi: 10.1002/advs.202507303 (PMC12631904; doi:10.1002/advs.202507303)
Supplement: Supplementary file 1 — Supporting Information [file ADVS-12-e07303-s003.pdf]

Supporting Information

Operando TEM Study of Partial Oxidation of Methane over Pd Nanoparticles

*Yingying Jiang[1, 2], Parinya (Lewis) Tangpakonsab[3], Alexander Genest[3], Günther Rupprechter[3], and Utkur Mirsaidov[1, 2, 4, 5]**

1. Department of Physics, National University of Singapore, Singapore, 117551, Singapore
2. Centre for BioImaging Sciences, Department of Biological Sciences, National University of Singapore, Singapore, 117557, Singapore
3. Institute of Materials Chemistry, TU Wien, 1060 Vienna, Austria
4. Centre for Advanced 2D Materials and Graphene Research Centre, National University of Singapore, Singapore, 117546, Singapore
5. Department of Materials Science and Engineering, National University of Singapore, Singapore, 117575, Singapore

*Correspondence: mirsaidov@nus.edu.sg

Table of Contents

1. Mass spectroscopy 1
2. Pd NPs during the POM reactions 3
3. DFT computations 5
4. Supporting video captions 9
5. Supporting references 9

1. Mass spectroscopy

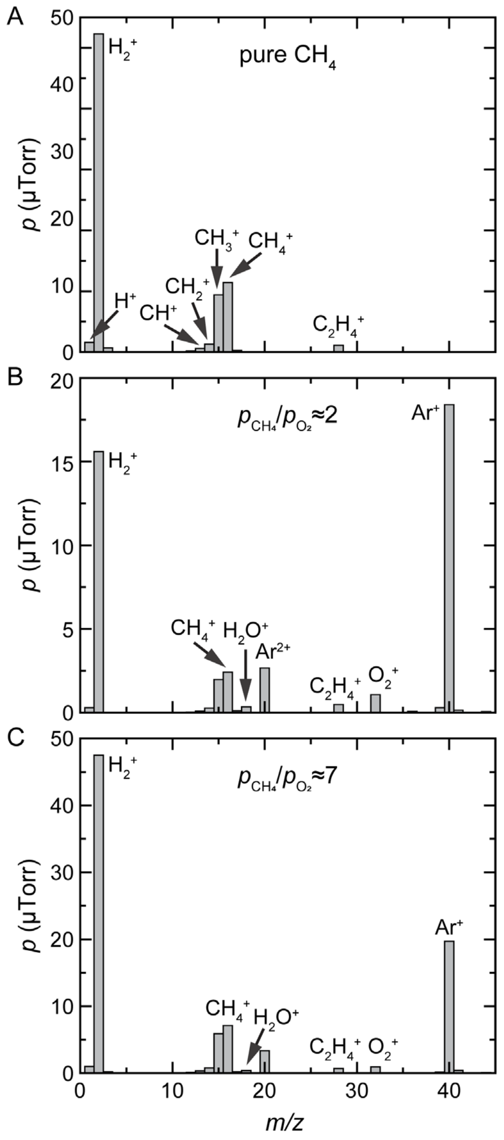

Figure S1. Mass spectra of pure CH_4 and the reactive gases. Mass spectra of (**A**) pure CH_4, and the reactive gases at (**B**) $p_{CH_4}/p_{O_2} \approx 2$ (760 Torr of 11% CH_4, 5% O_2, and 84% Ar) and (**C**) $p_{CH_4}/p_{O_2} \approx 7$ (760 Torr of 26% CH_4, 4% O_2, and 70% Ar).

In a mass spectrometer, neutral gas molecules are ionized into charged species for analysis, allowing the measurement of their mass-to-charge ratio (m/z).[1] Figure S1A shows the mass spectrum of pure CH_4, with peaks corresponding to its ionic species:[1-2]

$$CH_4 + e^- \rightarrow CH_4^+, CH_3^+, CH_2^+, CH^+, C^+, H_2^+, H^+ \quad (S1)$$

$$CH_4 + CH_2^+ \rightarrow C_2H_4^+ + H_2 \quad (S2)$$

The formation of $C_2H_4^+$ ($m/z = 28$) and H_2^+ ions contribute to the high baseline signals of CO^+ ($m/z = 28$) and H_2^+ in the mass spectra, as observed in Figures 1C, 1D, 3C, and 5B.

Figures S1B–C show the mass spectra of the reactive gases at $p_{CH_4}/p_{O_2} \approx 2$ and $p_{CH_4}/p_{O_2} \approx 7$, respectively. In addition to the ionic species of CH_4 (Equations S1–S2), O_2 ($O_2{}^+$), and Ar (Ar^+ and Ar^{2+}),[2] the spectra also reveal the presence of H_2O^+ ions ($m/z = 18$). These H_2O^+ ions are likely formed in the mass spectrometer via reactions between $H_2{}^+$ and O_2 or H_2 and $O_2{}^+$, and their formation contributes to the elevated baselines of H_2O^+ in Figures 1C, 1D, 3C, and 5B.

2. Pd NPs during the POM reactions

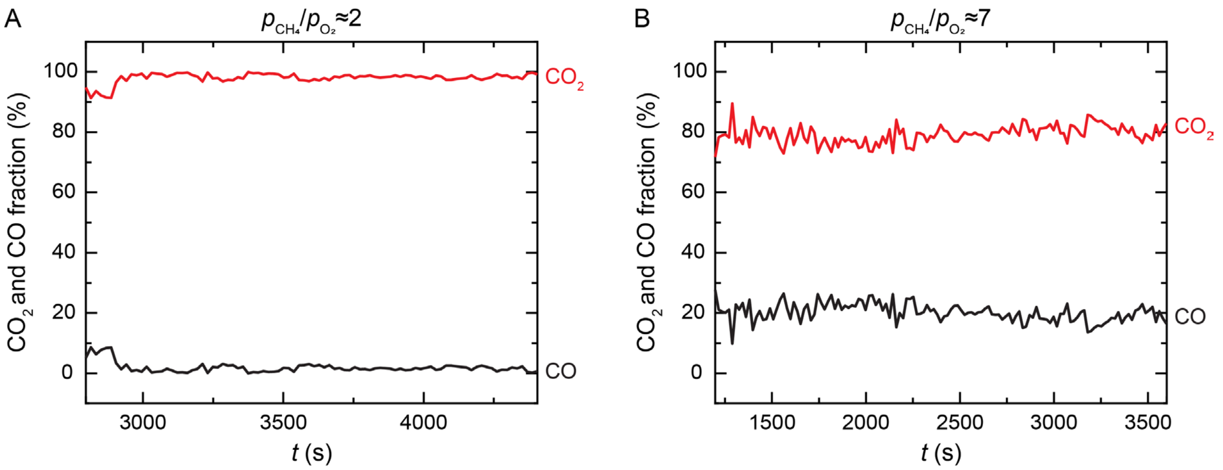

Figure S2. Amount of CO_2 and CO at different partial pressures. Estimated percentages of CO_2 and CO corresponding to the profiles shown in (**A**) Figure 1C and (**B**) Figure 1D (Materials and Methods describe how these values were estimated).

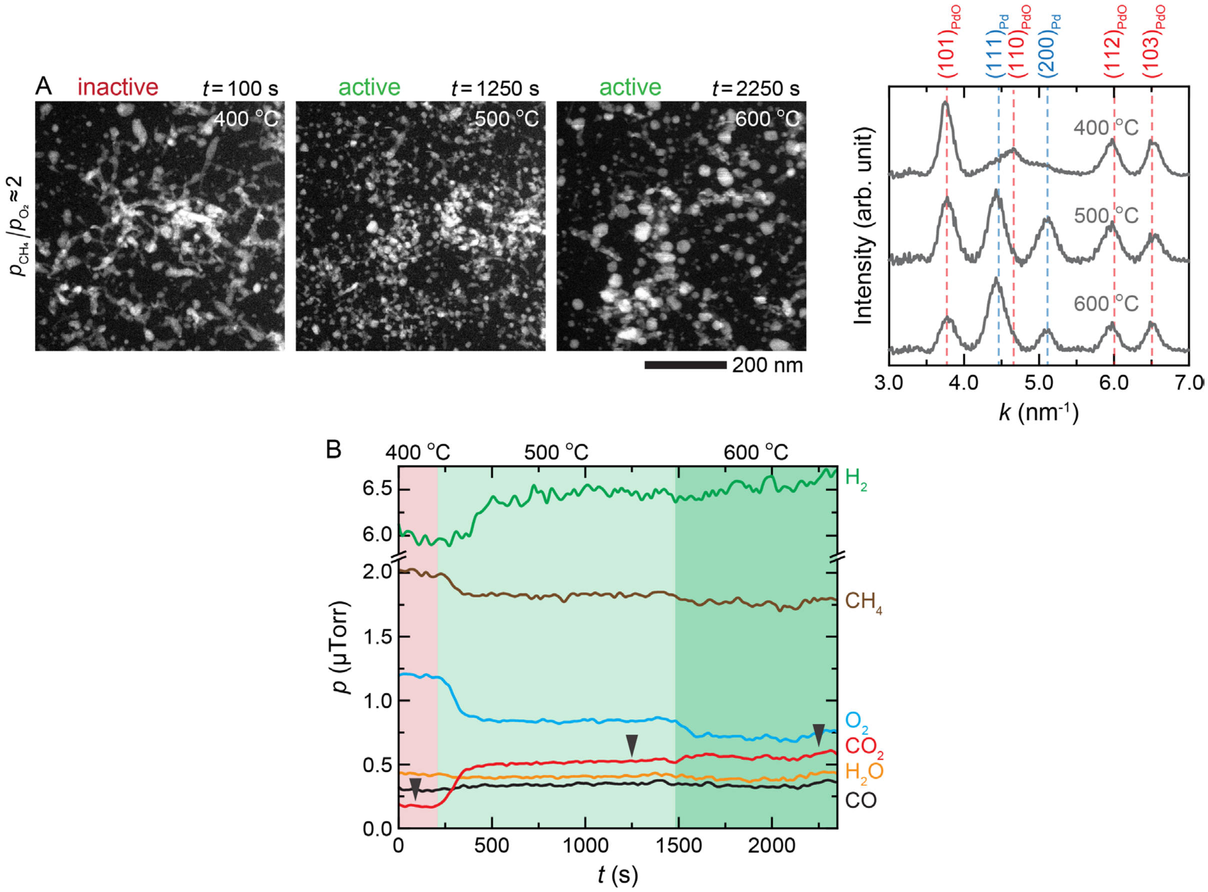

Figure S3. Pd–PdO NPs in the POM reaction at $p_{CH_4}/p_{O_2} \approx 2$. (**A**) STEM images of the Pd–PdO NPs, electron diffraction profiles, and (**B**) corresponding changes in the amount of reactants and products during the POM reaction at $p_{CH_4}/p_{O_2} \approx 2$ (760 Torr of 7% CH_4, 4% O_2, and 89% Ar). The dashed vertical lines in the diffraction profiles correspond to the diffraction peaks of Pd (blue) and PdO (red). The black arrows in (B) correspond to the time points of the STEM image series and electron diffraction profiles shown in (A). The results show that the Pd–PdO NPs have high catalytic activity at 500–600 °C.

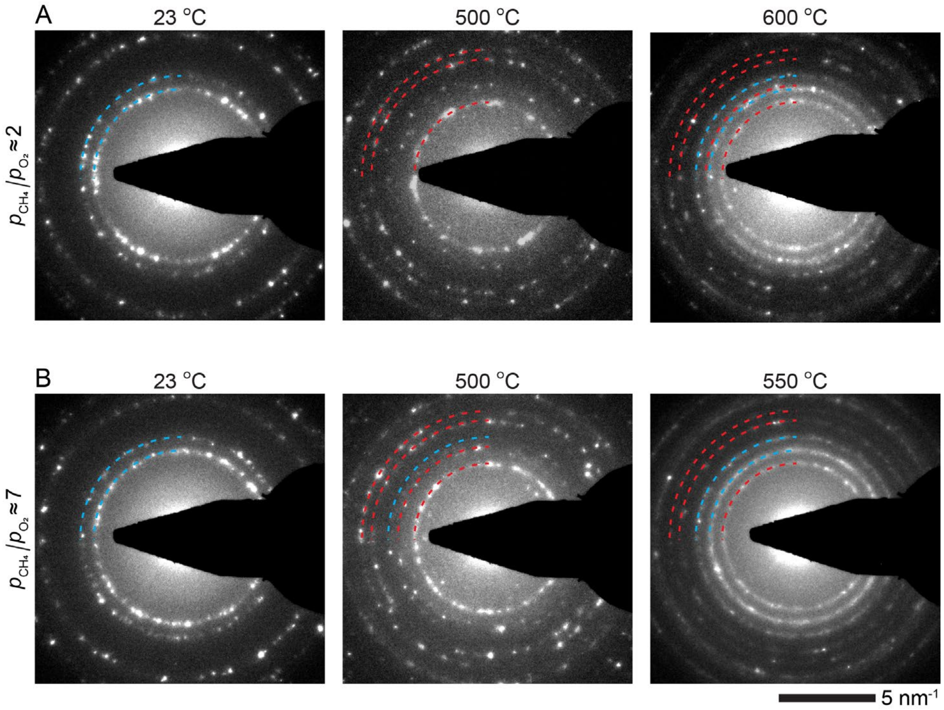

Figure S4. Electron diffraction patterns. Set of diffraction images corresponding to the diffraction profiles shown in (**A**) Figure 1A and (**B**) Figure 1B.

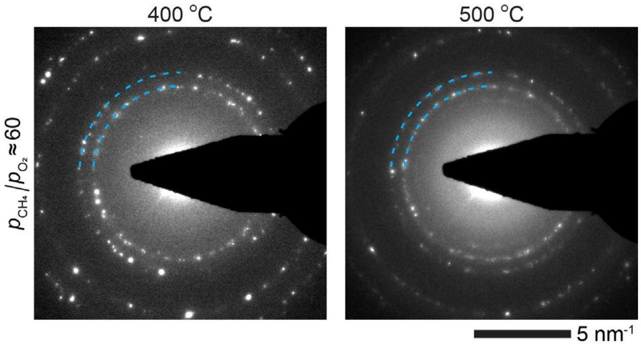

Figure S5. Electron diffraction patterns. Set of diffraction images corresponding to the diffraction profiles shown in Figure 3D.

3. DFT computations

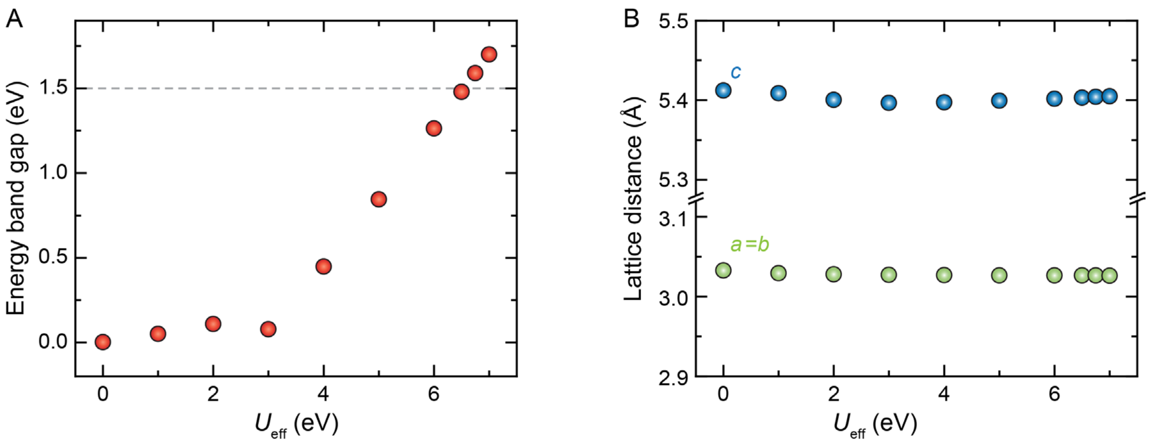

Figure S6. Effects of the effective *U* parameter on the energy band gap and lattice constant of bulk PdO. (**A**) Energy band gap and (**B**) lattice constants of bulk PdO as functions of the U_{eff}. At $U_{\mathrm{eff}} = 6.5$ eV, the calculated energy band gap of 1.48 eV closely matches the experimental value of 1.50 eV (dashed gray line).[3] The lattice constant of PdO remains nearly unchanged across different U_{eff} values (*i.e.*, $a = b = 3.03$ Å, $c = 5.40$ Å) and aligns closely with the experimental data (*i.e.*, $a = b = 3.03$ Å, $c = 5.33$ Å).[4] Consequently, $U_{\mathrm{eff}} = 6.5$ eV was chosen for all Pd atoms in the PdO region of the Pd–PdO interface model.

Table S1. Bader charge analysis of bulk Pd, PdO, and Pd_2O. The Bader charge q was analyzed for bulk Pd, PdO, and Pd_2O to identify the oxidation states of Pd species: Pd^0, Pd^{2+}, and Pd^{1+}.[5-7]

System	q_{Pd} (e^-)	q_{O} (e^-)	Pd^x
Pd (face-centered cubic)	10	–	Pd^0
PdO (tetragonal)	9.15	6.85	Pd^{2+}
Pd_2O (cubic)	9.59	6.82	Pd^{1+}

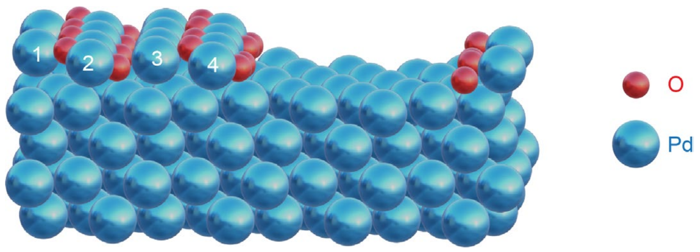

Figure S7. Pd atoms selected for charge analysis on the Pd–PdO interface model. The figure highlights four Pd columns (labelled 1–4) within the PdO region and the remaining Pd atoms in the model. These were analysed for their charge distribution, as detailed in Table S2.

Table S2. Charge analysis of the Pd–PdO interface model shown in Figure S7. The PdO region consists of Pd^{1+} (9.50 e^-) and Pd^{2+} (9.18 e^-), as determined by Bader charge analysis and the O nearest-neighbor (O NN). The Pd region has an average charge of 9.97 e^-, indicating the metal species is Pd^0.

Pd column	O NN	q_{Pd} (e^-)	Pd^x
1	2	9.50	Pd^{1+}
2	4	9.18	Pd^{2+}
3	2	9.50	Pd^{1+}
4	4	9.18	Pd^{2+}
others	–	9.97	Pd^0

Table S3. Adsorption energies of gas molecules and radicals on the Pd–PdO interface model. Adsorption energies (E_{ads}) and adsorption free energies (G_{ads}) of various gas molecules and radicals on the Pd region of the interface model (*i.e.*, site D in Figure 4A) were calculated using the PBE+*U*-D3 method. For comparison, the E_{ads} values for the pure Pd(100) surface (without PdO) were obtained using the standard PBE method from ref.[8]. The inclusion of the van der Waals (vdW) interaction in the DFT-D3 method contributes more than 0.2 eV ($|E_{\mathrm{difference}}| > 0.2$ eV) to the adsorption energies of these species.[9]

Species	E_{ads}(0 K) (eV)			G_{ads}(800 K) (eV)
	PBE+*U*-D3	PBE[8]	$\lvert E_{\mathrm{difference}}\rvert$	PBE+*U*-D3
CH_4*	-0.62	-0.01	0.61	0.21
CH_3*	-2.33	-1.77	0.56	-1.03
CH_2*	-4.48	-4.16	0.32	-2.92
CH*	-6.88	-6.61	0.27	-5.40
C*	-8.13	-7.89	0.24	-6.91
CO*	-2.41	-1.88	0.53	-1.08
CO_2*	-0.66	–	–	0.51
½H_2*	-0.81	-2.75	1.94	-0.46

Table S4. Steps of the CH_4 oxidation reaction and the corresponding reaction energies (ΔE) and reaction free energies (ΔG). The reaction proceeds via the complete dehydrogenation of CH_4 to elemental C* (steps **1**→**6**) and the oxidation of C* to CO* (step **6**→**7**), followed by the formation of CO_2 (steps **7**→**10a**) or CO (steps **7**→**11b**).

Reaction	State	Reaction step	ΔE(0 K) (eV)	ΔG(800 K) (eV)
Dehydrogenation	**1**. Bare	–	0.00	0.00
	**2**. CH_4*	**1→2**	-0.62	0.21
	**3**. CH_3* + H*	**2→3**	0.24	0.19
	**4**. CH_2* + 2H*	**3→4**	-0.12	-0.21
	**5**. CH* + 3H*	**4→5**	-0.68	-0.92
	**6**. C* + 4H*	**5→6**	-0.67	-0.92
	**7**. CO* + V_O + 4H*	**6→7**	-1.26	-1.50
Formation of CO_2	**8a**. CO* + ½O_2* +4H*	**7→8a**	-0.79	0.69
	**9a**. CO_2* +4H*	**8a→9a**	-2.59	-2.58
	**10a**. CO_2(g) + 4H*	**9a→10a**	0.66	-0.51
Formation of CO	**8b**. CO(g) + V_O + 4H*	**7→8b**	2.06	0.77
	**9b**. C* + ½O_2* + 8H*	½O_2* + 4H*+ CH_4*→**9b**[×]	-1.94	-1.62
	**10b**. CO* + 8H*	**9b→10b**	-3.76	-3.78
	**11b**. CO(g) + 8H*	**10b→11b**	2.41	1.08

[×] In step **8b→9b**, the dehydrogenation processes of CH_4 (steps **1→6**) were omitted, starting from the formation of C*. The total energy of ½O_2* + 4H*+ CH_4* was used as a reference for the calculations of ΔE and ΔG in this step.

Table S5. Activation energies (E_a) and activation free energies (G_a) at the transition states (TS).

TS	E_a (eV)	G_a (eV)
**(2→3)**	0.60	0.70
**(3→4)**	0.52	0.55
**(4→5)**	0.67	0.61
**(5→6)**	0.49	0.39
**(6→7)**	1.31	1.18
**(8a→9a)**	0.35	0.46
**(9b→10b)**	0.22	0.23

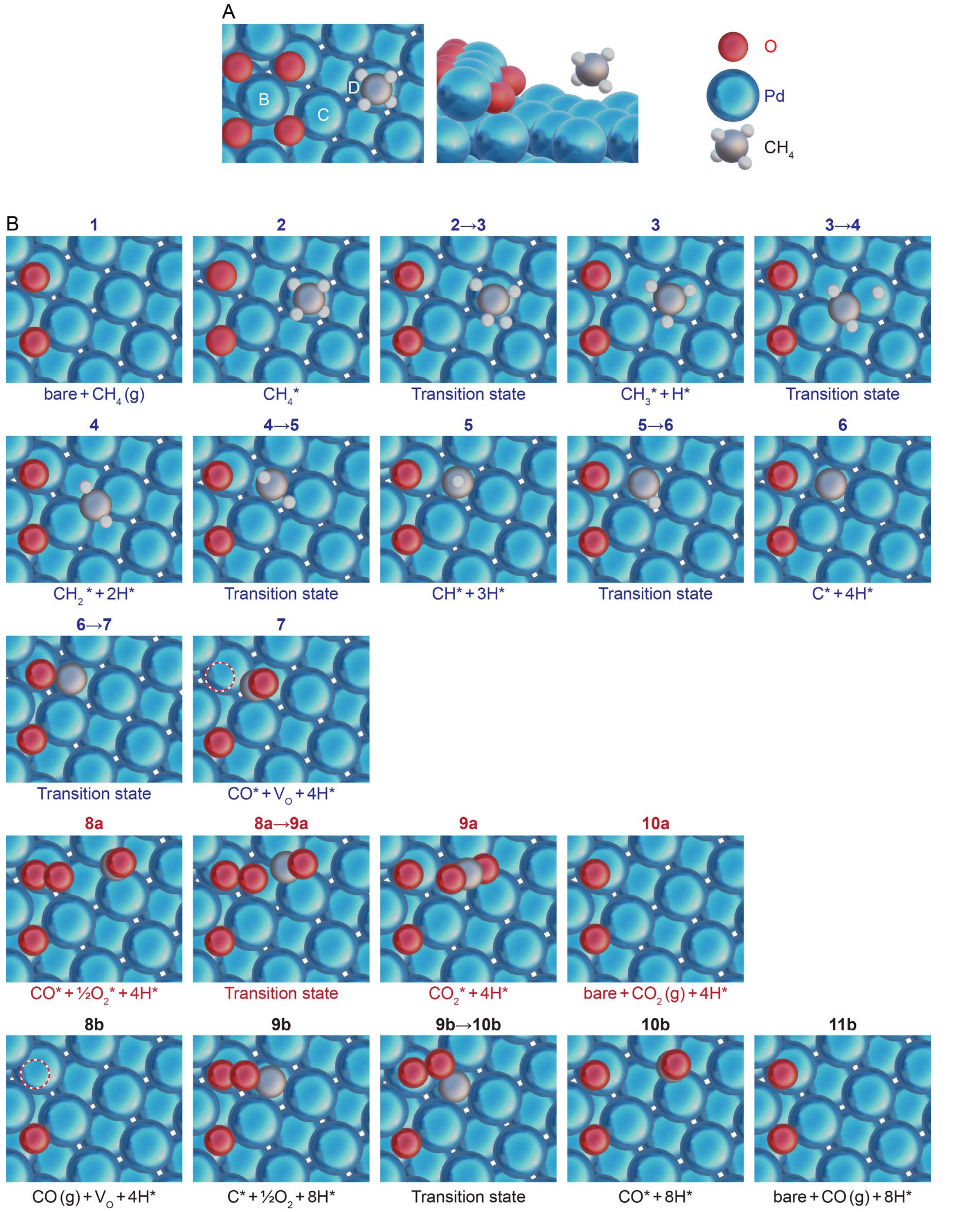

Figure S8. Atomic configurations of the CH_4 oxidation reaction on the Pd–PdO interface. (**A**) Adsorption of CH_4 on site D of the Pd–PdO interface. (**B**) CH_4 oxidation reaction on the Pd–PdO interface corresponding to Figure 4C.

4. Supporting video captions

Supporting Video 1: Fragmentation of the Pd NPs during the POM reaction at $p_{CH_4}/p_{O_2} \approx 7$ (760 Torr of 22% CH_4, 3% O_2, and 75% Ar) and at 500 °C, as shown in Figure 2A.

Supporting Video 2: Restructuring of the Pd NPs during the POM reaction at $p_{CH_4}/p_{O_2} \approx 7$ (760 Torr of 22% CH_4, 3% O_2, and 75% Ar) and at 500 °C, as shown in Figure 2C.

Supporting Video 3: Activation of the Pd NPs during the POM reaction at $p_{CH_4}/p_{O_2} \approx 60$ (760 Torr of 60% CH_4, 1% O_2, and 39% Ar) and at 400–500 °C, as shown in Figures 3A–C.

5. Supporting references

[1] J. H. Gross. in *Mass Spectrometry: A Textbook* (Springer, Cham, Switzerland, 2017).

[2] W. E. Wallace. Mass Spectra. In: *NIST Chemistry WebBook, NIST Standard Reference Database Number 69* (eds P. J. Linstrom, W. G. Mallard). National Institute of Standards and Technology, Gaithersburg, MD, 20899, https://doi.org/10.18434/T4D303, (retrieved June 9, 2024).

[3] H. Okamoto, T. Asô, Formation of thin films of PdO and their electric properties, *Jpn. J. Appl. Phys.* 1967, **6** (6), 779.

[4] J. Waser, H. A. Levy, S. W. Peterson, The structure of PdO, *Acta Crystallogr.* 1953, **6** (7), 661–663.

[5] W. Tang, E. Sanville, G. Henkelman, A grid-based Bader analysis algorithm without lattice bias, *J. Phys. Condens. Matter* 2009, **21** (8), 084204.

[6] E. Sanville, S. D. Kenny, R. Smith, G. Henkelman, Improved grid-based algorithm for Bader charge allocation, *J. Comput. Chem.* 2007, **28** (5), 899–908.

[7] G. Henkelman, A. Arnaldsson, H. Jónsson, A fast and robust algorithm for Bader decomposition of charge density, *Comput. Mater. Sci.* 2006, **36** (3), 354–360.

[8] A. Trinchero, A. Hellman, H. Grönbeck, Methane oxidation over Pd and Pt studied by DFT and kinetic modeling, *Surf. Sci.* 2013, **616**, 206–213.

[9] F. Chiter, V. B. Nguyen, N. Tarrat, M. Benoit, H. Tang, C. Lacaze-Dufaure, Effect of van der Waals corrections on DFT-computed metallic surface properties, *Mater. Res. Express* 2016, **3** (4), 046501.
